# ‘Crossing borders’ in data standardisation: application of OMOP CDM in an international clinical trial network in precision cancer medicine

**DOI:** 10.2340/1651-226X.2026.45120

**Published:** 2026-02-23

**Authors:** Maria Martin Agudo, Henk van der Pol, Gabriel Bratseth Stav, Tina Kringelbach, Katarina Puco, Åsmund Flobak, Hans Gelderblom, Kjetil Taskén, Gro Live Fagereng, Eivind Hovig

**Affiliations:** a Institute for Cancer Research, Oslo University Hospital, Oslo, Norway; b Department of Medical Oncology, Leiden University Medical Center, Leiden, The Netherlands; c Mathematical Institute, Leiden University, Leiden, The Netherlands; d Department of Oncology, Rigshospitalet, Copenhagen University Hospital, Copenhagen, Denmark; e Department of Oncology, Trondheim University Hospital, Trondheim, Norway

**Keywords:** Precision cancer medicine (PCM) clinical trials, data sharing network, standardisation, OMOP Common Data Model (CDM), ETL pipeline, evidence generation

## Introduction

The PRIME-ROSE initiative is a European collaboration involving 28 countries and 11 national precision cancer medicine (PCM) trials that are ongoing or starting soon [1]. It combines data from trials with similar designs using an umbrella–basket approach and has shown that PCM is feasible and beneficial in European countries [2–4]. Patients with advanced cancer are enrolled into cohorts defined by tumour type, molecular alteration and assigned drug. However, recruitment is slow because these alterations are rare [2].

The PRIME-ROSE main objective is to demonstrate the effectiveness and safety of expanding the indication, and pooling trial data accelerates evidence generation [5].

Several approaches can be applied to standardise the structure of the incoming data within a common framework. Widely used strategies include HL7 Fast Healthcare Interoperability Resources [6], Phenopackets [7] or the Observational Medical Outcomes Partnership (OMOP) Common Data Model (CDM) [8, 9]. The PRIME-ROSE consortium has adopted the OMOP CDM because it reduces variation across multisite data and supports the generation of reliable evidence [9–10, 11,12] in life sciences.

OMOP CDM allows harmonisation of the data from the different PCM clinical trials and retains the original values in the dedicated source fields. Additionally, the standardisation to OMOP CDM supports the usage of various Observational Health Data Sciences and Informatics (OHDSI) tools such as Usagi [13] for semantic mapping or Data Quality Dashboard (DQD) [14, 15] for quality check. Lastly, the standardisation to the OMOP CDM enables federated analysis in large precision oncology networks [16, 17].

The aim is to build an automated extract, transform and load (ETL) pipeline for rapid extraction of statistical outcomes from standardised, aggregated cohort patient data. PRIME-ROSE aims to establish a blueprint for sharing and pooling data between PCM trials.

## Methods

### Data sharing

Clinical trial data from ongoing trials are uploaded to and shared in the Service for Sensitive Data (TSD) after cohorts are completed. TSD is a secure environment managed by the University of Oslo. The consortium partners have agreed on sharing 41 variables (Table S1) featuring primary and secondary endpoints including progression-free survival [18].

### ETL pipeline

**Figure 1:**
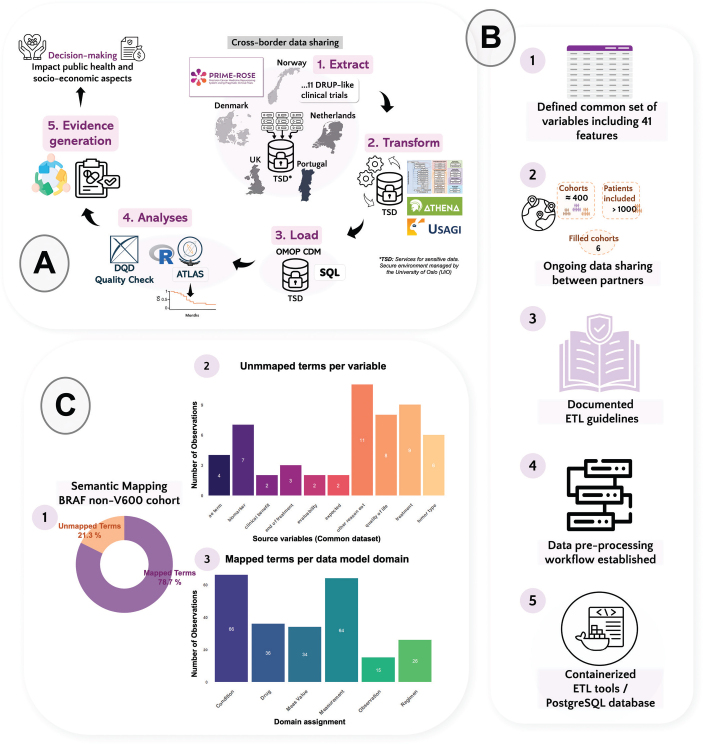
(A) The diagram shows the flow of the data within PRIME-ROSE. Sharing data between countries across Europe and the application of an extract, transform and load (ETL) process for data transformation and statistical analyses are key elements to accelerate the analysis of clinical trial data and evidence generation by enabling rapid patient cohort enrolment. (B) Summary with the major advances of the ETL development in PRIME-ROSE. The common dataset is only shared once the patient cohorts are filled (1 and 2). The ETL logic (3) guides the coding process for transforming the data and connecting the variables to the CDM Database. Currently, a pre-processing step prepares the data acquired from multiple sites (4). A minimal test for setting up software containers in the sensitive area TSD has been developed (5). (C) Semantic mapping was tested with the OHDSI tool Usagi in a BRAF-nonV600 cohort. (1) 306 source observations (terms) were analysed with Usagi and 78.7% (241) were mapped to a standard concept within the OHDSI standardised vocabulary catalogue. (2) The unmapped terms are confided to one of the nine variables displayed in the x axis of the bar plot, refining the semantic mapping process is crucial for the study. (3) Majority of the mapped terms connects to condition_ocurrence or measurement tables within the OMOP CDM version 5.4.

Data controllers of each trial submit the respective datasets according to the variables included in the common dataset. Subsequently, these data will be standardised to the OMOP CDM v5.4 using the ETL pipeline (see Figure 1A and Supplementary Material for a detailed description of the pipeline).

### ETL implementation and deployment

A pre-processing workflow was developed for sources where eCRF variables must be combined in datasets at the trial level. All records are validated against an internal schema and loaded into a harmonised data model. This ensures that downstream modules performing semantic and structural mapping to the OMOP CDM v5.4 target schema receive a consistent input.

For execution within the secure TSD environment, a prototype container-based deployment was tested, comprising a PostgreSQL database container (v15.0) and a separate ETL container with the Python application. Container images were built locally using Docker (v27.5.0) and executed using Podman (v5.6.0) [19].

## Results

### Common PRIME-ROSE variables

To date, the ongoing data sharing and manual aggregation between the different partners in PRIME-ROSE has led to merging of 396 cohorts with 1133 patients included. Six of the cohorts have been completed, and none has been stopped due to lack of efficacy (Figure 1B). A common dataset with 41 variables has been defined (Table S1).

### Mapping to OMOP CDM version 5.4

A first iteration of the ETL logic serves as a reference for mapping the PRIME-ROSE source variables into the OMOP CDM v5.4 structured tables (Figure 1B). Similarly as reported by Ajmal et al. [17], we found that for some of the common PRIME-ROSE variables (e.g. *Concomitant medication* or *Dose delivered)* there is no straightforward mapping to the model and additional relationships should be established.

### Semantic mapping challenges

Usagi (v1.4.3) was used to perform a semantic mapping test where source terms from aggregated data were mapped into standard concepts from the OHDSI standardised vocabularies (v5.0 27-FEB-25) (see Table S2). A BRAF-nonV600 cohort (Figure 1C) was selected, consisting of 53 patients recruited in three trials: (1) DRUP (Netherlands), (2) IMPRESS-Norway (Norway), and (3) FINPROVE (Finland).

In total, 306 source terms were processed. From those, 78.7% (241) were successfully mapped (Figure 1C1) to a standard concept within one of the six domains in Figure 1C3. Mostly, the mapped terms belonged to condition_ocurrence or measurement tables within the OMOP CDM v5.4. As shown in Figure 1C1, 21.3% of the terms (*n*=65) remained unmapped, which belonged to one of the nine variables displayed in the plot (Figure 1C2). This can happen in variables where the input is free-text and it contains a misspelling, as seen in one observation for our aggregated dataset. Also, we found that broad terms are employed for defining some of the biomarkers/targets, for example, fusions or BRAF activating mutations. These are not specifically mapped to the OHDSI vocabulary, OMOP Genomic, which contains 289889 concepts. Finally, most of the unmapped terms belonged to the free-text variable *other reasons for end of treatment (eot).*

## Discussion and conclusion

Developing an ETL, such as the one presented here, is a dynamic and malleable process that should accommodate the different needs of the partners who are sharing the data. To ease versioning, reproducibility, stabilisation and individualisation of processes, we utilise Docker containers. From our experience, semantic mapping of certain variables such as *biomarker* or even *adverse events* is complex, and probably requires a higher level of data granularity or standard terms to input in the mapping and/or more extensive vocabularies (e.g. more concepts in OMOP genomics OHDSI vocabulary). Also, other variables with free-text observations such as *other reasons end of treatment* are difficult to map, as textual similarity comparisons with Usagi has some limitations. We are refining the process by performing data validation, maintaining multidisciplinary collaboration with experts and investigating alternative approaches, including machine learning-based tools to enhance semantic mapping.

PRIME-ROSE implements FAIR (Findable, Accessible, Interoperable, and Reusable) principles [20]. We are commited to open science, within the limits of sensitive patient data protection, as much as possible, in order to benefit the scientific and public community. FAIRification and standardisation to OMOP CDM prepares PRIME-ROSE for the implementation of the European Health Data Space (EHDS) [21, 22], as a key ecosystem to harbour large-scale evidence networks such as EHDEN [10].

This work with ongoing PCM trials in Europe showcases how standardised and structured data in PCM may facilitate cross-border data sharing to influence the development of PCM, particularly for rare cancer trials. It can serve as a blueprint for similar or expanded initiatives such as Joint Action on Personalised Cancer Medicine (JA PCM) [23]. PRIME-ROSE aims to increase the number of partners, and implementation of standardisation to OMOP CDM is key to enable federated analysis in PCM with similar trials outside of the European consortium.

## Supplementary Material



## Data Availability

The methodology described in this short report has been developed and tested using: (i) a synthetic dataset created for pipeline development purposes, and (ii) a small patient cohort dataset. Relevant code is publicly available in the GitHub repository https://github.com/pcm-primerose/omop_etl. However, for ethical reasons and in complicance with European GDPR regulations, to protect the patient information, datasets are not publicly available.
