# Supplementary material for: ‘Crossing borders’ in data standardisation: application of OMOP CDM in an international clinical trial network in precision cancer medicine
Source: Acta Oncol. 2026 Feb 23;65:45120. doi: 10.2340/1651-226X.2026.45120 (PMC12946775; doi:10.2340/1651-226X.2026.45120)
Supplement: Supplementary file 1 [file AO-65-45120-s1.pdf]

Supplementary material has been published as submitted. It has not been copyedited, or typeset by Acta Oncologica

Supplementary material

Methods

ETL pipeline

Pipeline steps are as follows:

i) Pre-processing data across trials: This part involves processes such as variable renaming, standardisation of date formats, handling of missing values or verification of data types (e.g., numeric vs integer).

ii) Data transformation: Source observations are translated into concepts from the corresponding OHDSI standardised vocabulary [1]. For example, the study drug at ingredient level trametinib will be mapped to a concept within the RxNorm vocabulary as available in the OHDSI vocabulary repository Athena. For this step, the OHDSI tool Usagi (v1.4.3) is utilised to make suggested mappings based on textual similarity of code descriptions. Then, these are loaded into the different tables of the OMOP CDM database following a specific logic designed for the PRIME-ROSE project.

iii) Data quality control: The OHDSI tool Data Quality Dashboard (DQD) will be included in the ETL, and the data quality will be assessed after the transformation, similar to previous studies [2,3]. DQD runs multiple checks, including verification of each table in the CDM to ensure that the fields are present as expected [2].

iv) Cohort analysis: Using the shared endpoints, the harmonised endpoints are evaluated. These include Clinical Benefit after 16 weeks of treatment, treatment-related adverse events, Overall Survival, Progression-Free Survival, and health-related Quality of Life outcomes. In alignment with this work, custom R Statistical Software scripts [4] for the analysis of key clinical outcomes are developed and implemented in the pipeline, to generate robust and reproducible evidence. Also, additional analyses are performed with the OHDSI tool ATLAS. The tool enables cohort definition and visualisation of the different characteristics of the patient cohort (e.g., drug treatment in a given period of time).

References:

1. Reich C, Ostropolets A, Ryan P, Rijnbeek P, Schuemie M, Davydov A, et al. OHDSI Standardized Vocabularies—a large-scale centralized reference ontology for international data harmonization. J Am Med Inform Assoc. 2024 Feb 16;31(3):583–90. https://doi.org/10.1093/jamia/ocad247.

2. Blacketer C, Voss EA, DeFalco F, Hughes N, Schuemie MJ, Moinat M, et al. Using the Data Quality Dashboard to Improve the EHDEN Network. Appl Sci. 2021 Dec 15;11(24):11920. https://doi.org/10.3390/app112411920.

3. Trinh NT, Houghtaling J, Bernal FL, Hayati S, Maglanoc LA, Lupattelli A, et al. Harmonizing Norwegian registries onto OMOP common data model: Mapping challenges and opportunities for pregnancy and COVID-19 research. Int J Med Inf. 2024 Nov;191:105602. https://doi.org/10.1016/j.ijmedinf.2024.105602.

4. R Core Team. R Core Team (2021). R: A language and environment for statistical computing. R Foundation for Statistical Computing, Vienna, Austria. URL https://www.R-project.org/. 2021.

Tables

Table S1: **List of common PRIME-ROSE variables shared by the different precision cancer medicine (PCM) clinical trials.** The variables are only shared when the patient cohorts are completed. The table contains primary variables, and some of them are generated using multiple secondary variables, e.g., Dose delivered requires to retrieve information regarding the administration of the treatment (oral or intravenous), discontinuation, etc...In the first iteration of the extract, transform and load (ETL) structural mapping logic, the primary and secondary variables have been mapped to the different tables of the OMOP Common Data Model version 5.4 (column OMOP CDM v5.4). (*) Dates connected to other variables.

	VARIABLE	DESCRIPTION	OMOP CDM v5.4
PERSON	Cohort Name	Target/Tumor Type/Treatment	Observation
	Trial	Name of the Trial	Observation
	ID	ID based on trial of origin	Person
	Tumor type	IDC10 / Tumor Type given each trial list	Condition_ocurrence
	Study drug 1	Study drug given to patient	Drug_exposure
	Study drug 2	Study drug given to patient in combination with Study Drug 1 (if applicable)	Drug_exposure
	Biomarker/Target	Biomarker name as per trial list	Measurement
	Biomarker details	Molecular signature (alteration). Gene or protein level	Measurement
	Age	Age at treatment start	Person
	Sex	Biological sex (male/female)	Person
	ECOG/WHO performance status	At baseline	Measurement
	Death	Date of death	Death
TREATMENT INFORMATION	***Previous treatment lines***	Which treatment lines were undertaken	Procedure_ocurrence
	Lost to follow-up	Date lost to follow-up	Observation
	Evaluability	If patient is evaluable for efficacy analysis (Yes/No)	Observation
	Treatment start	First dose in first cycle (date)	Drug_exposure
	Treatment start cycle	First day of treatment cycle start (date)	Drug_exposure
	Treatment end cycle	Date of treatment cycle end (date)	Drug_exposure
	Treatment start last cycle	First day in last treatment cycle (date)	Drug_exposure
	Treatment end	Date for disease progression and last dose	Drug_exposure
	Dose delivered	Dose delivered/cycle including prescribed dose and unit	Drug_exposure
SAFETY	Concomitant medication	All concomitant medication	Drug_exposure
	Adverse Event (AE)	Has the patient had any Adverse Events? (Yes/No)	Observation
	AE grade	Worst CTCAE severity grade of the Adverse Event (grade 3 and higher)	Observation
	AE CTCAE Term	The adverse event term (CATCAE Terminology)	Condition_ocurrence
	AE start date	AE start date (date)	Condition_ocurrence*
	AE end date	AE end date (date)	Condition_ocurrence*
	AE outcome	What was the outcome of this AE?	Observation
	Number of AEs	Total number of AEs the patient has experienced	Measurement

	SAE	Was the Adverse Event (AE) serious (SAE)? (Yes/No)	Observation
	Related to Treatment	Is the AE related to the study treatment?	Observation
	Expectedness	Suspected Unexpected Serious Adverse Reaction (SUSAR) (specify drug)	Observation
EFFECT	Type tumor assessment	Type of tumor assessment (RECIST, iRECIST, LUAGNO, RANO, AML)	Measurement
	Event date assessment	Date for tumor assessment (date)	Observation
	Baseline evaluation	Sum Size of target lesion at baseline, non-target-lesions (Yes/No)	Measurement
	Change from baseline	Sum size of target lesion at visit, % change from baseline, evaluation non-target-lesion, new lesions?	Measurement
	Response assessment	Response assessment from type tumor assessment	Measurement
	Reason end of treatment (EOT)	The reason for EOT: PD, not evaluated, death, etc.	Observation
	Best overall response (BOR)	Confirmed best overall response	Observation
	Clinical benefit (CB)	CR, PR or SD after 16 weeks of treatment	Observation
	Quality of life (QoL) assessment	Health-related Quality of Life (HrQoL)-questionnaires collected in the trial (e.g., QLQ-C30 or EQ-5D-5L)	Measurement

Table S2: **Terms mapped to any of the OHDSI standardised vocabularies**. During the first iteration of the semantic mapping, terms were mapped to eight different standard vocabularies, including RxNorm that provides normalised names for clinical drugs.

	VARIABLE	***MAPPED VOCABULARY***
DEMOGRAPHIC	Tumor type	*SNOMED*
	Study drug 1	*RxNorm*
	Study drug 2	*RxNorm*
	Biomarker/Target	*OMOP Genomic*
	Biomarker details	*OMOP Genomic*
	Sex	*LOINC*
	ECOG/WHO performance status	*SNOMED*
TREATMENT INFORMATION	Previous treatment lines	*RxNorm / RxNorm Extension / HemOnc*
SAFETY	AE grade	*SNOMED*
	AE CTCAE Term	*SNOMED*
	Related to Treatment	*LOINC*
EFFECT	Reason end of treatment (EOT)	*LOINC / SNOMED*
	Best overall response (BOR)	*LOINC / NAACCR*
	Quality of life (QoL) assessment	*SNOMED / OMOP Extension*
